# Integrated Glycosylation Patterns of Glycoproteins and DNA Methylation Landscapes in Mammalian Oogenesis and Preimplantation Embryo Development

**DOI:** 10.3389/fcell.2020.00555

**Published:** 2020-07-10

**Authors:** Jian Wang, Geng G. Tian, Xiaoyong Li, Yangyang Sun, Li Cheng, Yanfei Li, Yue Shen, Xuejin Chen, Wenwei Tang, Shengce Tao, Ji Wu

**Affiliations:** ^1^Renji Hospital, Key Laboratory for the Genetics of Developmental and Neuropsychiatric Disorders (Ministry of Education), Bio-X Institutes, School of Medicine, Shanghai Jiao Tong University, Shanghai, China; ^2^Shanghai Center for Systems Biomedicine, Key Laboratory of Systems Biomedicine (Ministry of Education), Shanghai Jiao Tong University, Shanghai, China; ^3^Key Laboratory of Fertility Preservation and Maintenance of Ministry of Education, Ningxia Medical University, Yinchuan, China; ^4^Department of Laboratory Animal Sciences, School of Medicine, Shanghai Jiao Tong University, Shanghai, China; ^5^School of Chemistry Science and Technology, Shanghai Key Laboratory of Chemical Assessment and Sustainability, Tongji University, Shanghai, China

**Keywords:** glycosylation, preimplantation embryo, lectin microarray, electrochemical technique, DNA methylation

## Abstract

Glycosylation is one of the most fundamental post-translational modifications. However, the glycosylation patterns of glycoproteins have not been analyzed in mammalian preimplantation embryos, because of technical difficulties and scarcity of the required materials. Using high-throughput lectin microarrays of low-input cells and electrochemical techniques, an integration analysis of the DNA methylation and glycosylation landscapes of mammal oogenesis and preimplantation embryo development was performed. Highly noticeable changes occurred in the level of protein glycosylation during these events. Further analysis identified several stage-specific lectins including LEL, MNA-M, and MAL I. It was later confirmed that LEL was involved in mammalian oogenesis and preimplantation embryogenesis, and might be a marker of FGSC differentiation. Modified nanocomposite polyaniline/AuNPs were characterized by electron microscopy and modification on bare gold electrodes using layer-by-layer assembly technology. These nanoparticles were further subjected to accuracy measurements by analyzing the protein level of ten-eleven translocation protein (TET), which is an important enzyme in DNA demethylation that is regulated by *O*-glycosylation. Subsequent results showed that the variations in the glycosylation patterns of glycoproteins were opposite to those of the TET levels. Moreover, analysis of correlation between the changes in glyco-gene expression and female germline stem cell glycosylation profiles indicated that glycosylation was related to DNA methylation. Subsequent integration analysis showed that the trend in the variations of glycosylation patterns of glycoproteins was similar to that of DNA methylation and opposite to that of the TET protein levels during female germ cell and preimplantation embryo development. Our findings provide insight into the complex molecular mechanisms that regulate human embryo development, and a foundation for further elucidation of early embryonic development and informed reproductive medicine.

## Introduction

Post-translational modification refers to the covalent processing of translated proteins. By adding modifying groups to one or more amino acid residues, the physical and chemical properties of the proteins can change and this can affect protein spatial conformation, active state, subcellular localization, folding, and stability, and protein–protein interactions (Abdrabou and Wang, 2018). A change in the abundance of protein post-translational modifications is of great significance, because abnormal post-translational modification can lead to a variety of diseases.

Glycosylation is one of the most important post-translational modifications. It is the process by which carbohydrate residues, or glycans, are attached to biomolecules to produce glycoconjugates, and is the primary form of protein and lipid post-translational modifications (Spiro, 2002). Glycoproteins are widely distributed in organisms and involved in the regulation of many biological processes, including mammal oogenesis and preimplantation embryo development (PED). Dehennaut et al. found that alloxan, an *O*-GlcNAc glycosyltransferase inhibitor, could inhibit G2/M phase transformation of oocytes (Dehennaut et al., 2007); they also found that *O*-GlcNAc glycosyltransferase inhibition can delay or block germinal vesicle breakdown (Dehennaut et al., 2008). A previous study showed that *N*-glycosylation of zona pellucida glycoproteins play an important role in sperm–egg binding (Lay et al., 2013). The stage-specific embryo antigens SSEA-1, SSEA-3, and SSEA-4 are all glycoproteins. Laminin is an *n*-acetylglucosylated polysaccharide protein that glycosylates at multiple sites and plays an important role in early embryonic development and organ formation. A previous study showed that glycosylation is also involved in regulation of polar surface structures and densification of blastocyst surfaces (Lee et al., 2016). Although the dynamic patterns of epigenetic regulation have been analyzed, including DNA methylation (Guo et al., 2014), histone modification (Liu et al., 2016), non-coding RNAs (Fan et al., 2015), and 3D chromatin structures (Ke et al., 2017), the glycosylation patterns of glycoproteins have not been investigated in mammalian oogenesis and preimplantation embryo development because of technical difficulties and the scarcity of required materials.

The ten-eleven translocation (TET) family of dioxygenases converts 5-methylcytosine to 5-hydroxymethylcytosine and provides a vital mechanism for DNA demethylation. Functional studies have revealed that TET-mediated DNA demethylation plays an important role in mouse oogenesis and embryonic development (Gu et al., 2011; Dai et al., 2016). Owing to the difficulty of accurately detecting TET protein level with a very small number of cells by conventional techniques, the dynamic patterns of TET in mammal oogenesis and preimplantation embryo development have not been analyzed. Fortunately, the development of electrochemical technology makes it possible to accurately measure TET protein content in a very small number of cells, because of its high sensitivity, low instrumentation cost, relatively shorter analysis time, and lack of tedious extraction procedures compared with other analytical techniques (Pandit et al., 2017).

Polyaniline (PANI), a conventional conducting polymer, is currently the most popular and considered the most promising sensing material because of its excellent environmental stability, high solution process ability, adequate conductance, and unique sensing function (Bai et al., 2015). Based on the above characteristics, the application of PANI onto suitable biosensors has been used to detect biological samples (Wang et al., 2014). Additionally, significant importance has been given to gold nanoparticles (AuNP) since their invention. AuNP possesses superior biocompatibility and conductivity, strong adsorption ability, and distinct physical and chemical attributes (Boisselier and Astruc, 2009). These properties make AuNP an excellent scaffold for producing novel chemical and biological sensors. Consequently, using PANI and AuNP enables detection of changes in levels of some important proteins, such as TET, during embryonic development from a small number of samples; these data can then be used for comparison of glycosylation patterns of glycoproteins and DNA methylation.

This study investigated the glycosylation patterns of glycoproteins and DNA methylation landscapes during mammalian oogenesis and preimplantation embryo development by high-throughput lectin microarray, electrochemical techniques, and genome-wide DNA methylation analysis. Our findings provide important integrated information about the glycosylation patterns of glycoproteins and DNA methylation during female germ cell and preimplantation embryo development. They also provide an insight into the field of glycomics that can be used for further study of associated molecular mechanisms.

## Materials and Methods

### Animals

The Stella-EGFP-IRES-Cre/Ert2 transgenic mice used in this experiment were donated by Professor Xingxu Huang, School of Life Sciences and Technology, Shanghai University of Science and Technology. SPF-grade wild C57BL/6 mice were purchased from Shanghai SLAC Laboratory Animal Co., Ltd. Animal experimentation was approved by the Institutional Animal Care and Use Committee of Shanghai and performed in accordance with the National Research Council Guide for Care and Use of Laboratory Animals.

### Mouse PGC Collection

To isolate mouse PGCs, 8–12 female Stella-Egfp-IRES-Cre/Ert2 transgenic mice genital ridges were isolated from 12.5-dpc (Days post coitum) female embryos and dissociated. Then, the dissected tissues were placed in RNase-free D-Hanks’ solution that contained collagenase IV (1 mg/ml, Sigma, United States), followed by incubation at 37°C with gentle agitation for approximately 40 min. The suspension was centrifuged at 300 *g* for 5 min, and the supernatant was carefully separated from the pellet. The pellet was resuspended, and clumps of cells were removed by passing the suspension through a 70-μm nylon cell strainer. The GFP-positive cells, 12.5-dpc PGCs, were obtained by flow cytometry.

### Mouse FGSC Collection

The mouse FGSC line was cultured *in vitro* according to our previous studies (Zou et al., 2009; Wu et al., 2017; Zhu et al., 2018). When the cell density of subculture reached 70–80%, trypsin was used to digest the cells into a single cell suspension, which was inoculated on a cell culture plate coated with gelatin and cultured in a 37°C, 5% CO_2_ incubator for 1 h. Then, the non-adherent suspension cells were transferred to a new gelatin-coated cell culture plate. To remove feeder cells, FGSCs were obtained by repeatedly sticking to the wall 3–4 times.

### Mouse Oocyte Collection

GV oocytes were collected from the ovaries of a 6-week-old C57BL/6 female mice 46–48 h after injection of 8–10 U pregnant mare serum gonadotropin (PMSG). The antral follicles were punctured by 30-gauge needles, and the GV oocytes enclosed by several layers of cumulus cells were selected. Adherent cumulus cells were removed by hyaluronidase treatment, and the denuded oocytes were collected. MII oocytes were collected from the oviducts of a 6-week-old C57BL/6 female mouse after sequential injection of PMSG and human chorionic gonadotropin. Adherent cumulus cells were removed by hyaluronidase treatment, and the cumulus cell-free MII oocytes were collected.

### Mouse Embryo Collection

For timed pregnancy, PMSG (8-10 UI) was intraperitoneally injected into C57BL/6 female mice aged 6–8 weeks. Next, hCG (8-10 UI) was intraperitoneally injected after 46–48 h. Pregnant mice were euthanized at various time points to obtain embryos as follows: zygote (21–25 h), 2-cell (45–48 h), 4-cell embryo (60 h), and blastocyst (3.5–4.5 dpc).

### Flow Cytometry and Cell Sorting

After MACS, the cells were suspended in PBS and subjected to flow cytometry to analyze and sort GFP-positive cells using a FACSAria II cell sorter equipped with BD software (Becton Dickinson).

### Sample Preparation and Lectin Microarray Analysis

The samples for the lectin microarray analysis were prepared by modifying a previously described method (Li et al., 2011a, b; Zhou et al., 2011; Xin et al., 2018). The samples were centrifuged at 350 *g* for 5 min to collect the cells and then washed with PBS. This is followed by fixation with 2% paraformaldehyde that containing 0.2% glutaraldehyde for 15 min. The samples were washed with PBS again before being stored at 4°C for subsequent lectin microarray. The preparation of lectin microarray was consistent with the previously reported (Li et al., 2011a). Simply, 56 lectins with concentrations of 1 μg/μl each were printed in triplicates on OPPolymer Slide H slides (Capital Bio, Beijing, China). They were stored overnight at 4°C to allow the lectins to be coated on the surface. Thus, the slides were ready for the samples detection.

Lectin microarray was first blocked in 10 mM Tris Buffered Saline with 0.5% (v/v) Tween-20 (TBST) for 1 h at RT and then washed once in PBST for 10 min, followed with twice in PBS for 10 min. The fixed sample labeled with propidine iodide (PI, 20 μg/ml) was adjusted concentration to 5 × 10^6^ cells in 200 μl PBS with 50 μM CaCl_2_ and 50 μM MnCl_2_ for each block of lectin microarray, and then incubated in a wet box for 1 h at RT in the dark. Each sample was repeated four times in and between slides with a diagonal manner. After the excess and unbound cells gently removed by submerging and inverting the slides in PBST, the air-dried slides were scanned with a GenePix 4200A (Molecular Devices, Sunnyvale, CA) at 5 μm resolution with the scanning condition set to 532 nm filter and 40% PMT value.

The scanning results of lectin microarray were analyzed by GenePix Pro 6.0. The lectin microarray experiments of each sample were repeated twice in biology and three times in technology. After the lectin microarray data of different samples were normalized by the maximum value, cluster analysis was carried out using the R package ggplots; the cluster method was “complete linkage,” and the PCA was carried out using the R package Rgl. Student’s *t*-test was used to analyze the differences of glycosylation patterns among different samples (the fluorescence signal intensity of lectin was ≥1.5; differences at *P* < 0.05 were considered significant).

### Glycosyltransferase Gene Expression Analysis

The transcriptome data of PGCs, FGSCs, GV oocytes, MII oocytes, fertilized oocytes, 2-cell embryos, 4-cell embryos, and blastocysts were downloaded from the Gene Expression Omnibus (GEO) and Sequence Read Archive (SRA) databases of NCBI GSE94136 (Miyauchi et al., 2017), SRP066132 (Zhang et al., 2016b), ERP00689 (Smallwood et al., 2011), GSE71434 (Zhang et al., 2016a), GSE70608 (Liu et al., 2016), GSE66582 (Wu et al., 2016). After data normalization, differential expression of the glycosyltransferase gene was analyzed.

### Lectin-Based Immunofluorescence Assays

Cells cultured in 48-well plates were washed with 1× phosphate-buffered saline (PBS), fixed in 4% formaldehyde for 30 min at room temperature, and then washed three times with PBS for 5 min each wash. Next, cells were permeabilized with 0.5% Triton X-100 for 30 min at room temperature, and then washed with PBS three times. Cells were then incubated at 37°C for 10 min in blocking buffer (PBS containing 10% goat serum). Next, the cells were incubated overnight at 4°C with the fluorescein LEL (1:500, vector lab). After washing three times with PBS, the cells were incubated at 37°C for 10 min with 500 ng/mL 4′,6-diamidino-2- phenylindole (DAPI; Sigma). Images were acquired using a Leica digital camera under a fluorescence microscope (DM2500, DMI3000B; Leica).

### Lectin-Based Immunosorbent Assays

Approximately 10 cells were used from each developmental stage to investigate Lycopersicon esculentum lectin level by lectin-based immunosorbent assays, developing lectin-based immunosorbent assays were performed as described in previous studies (Li et al., 2011a). In brief, 10 μL of each sample was added in triplicate into blocked buffer coated 384-MSD plates. Then 10 μL of 20 μg/mL biotinylated LEL were mixed with 5 μg/mL streptavidin SMLFO-TAG. Finally, 50 μL of 1 × MSD plate read buffer was added to each well and electrochemiluminescence was detected using the MSD SECTOR Imager 2400. The statistical analyses were done using Graphpad Prism V5 software. The significance was set at 0.05.

### Electrochemical Measurements

Approximately 200 cells were used from each developmental stage to investigate the TET protein level by electrochemical techniques. Sample preparation, electrochemical measurement, and data analysis were performed as described in previous studies (Ozcan et al., 2017; Pandit et al., 2017; Wang et al., 2017).

### Treatment With 5-aza-2′-Deoxycytidine

5-aza-2′-deoxycytidine (5-aza-2dC, Sigma-Aldrich) was dissolved in acetic acid: water (1:1) to 10 mM final concentration. Twenty-four hours after seeding, the cells were incubated for 72 h with 5-aza-2dC (1 μM). Every 24 h, the medium was changed to a fresh one containing the same concentration of the inhibitor. Control cells were treated with dissolving buffer.

### CpG Methylation Analysis by Pyrosequencing

To estimate genome-wide methylation, we analyzed the LINE-1 elements. Genomic DNA was extracted from FGSCs. For bisulfite sequencing analysis of methylation, 500 ng genomic DNA was processed using an EZ DNA Methylation-Gold Kit (ZYMO Research, United States) following the manufacturer’s instructions. The methylation status of LINE-1 was analyzed using specific primers (F: GGTTGAGGTAGTATTTTGTGTG, R: TCCAAAAACTATCAAATTCTCTAAC). PCR products were sequenced and CpG islands were analyzed.

### qRT-PCR

Total RNA from cells was isolated using Trizol reagent. Complementary DNA was synthesized from 2 μg total RNA using a High Capacity cDNA Reverse Transcription Kit (Invitrogen, United States). Primers were designed using Primer Premier 5.0. Primer details are listed in Supplementary Table S1. *Gapdh* was amplified in each sample as an internal control. The mRNA level of each gene was normalized to *Gapdh* expression. The specificity of all quantitative real-time PCRs was verified by a single peak in the melting curve. Quantitative real-time PCRs were performed with a 7500 real-time PCR amplification system using SYBR Green PCR Master Mix (Applied Biosystems, United Kingdom). The relative transcript levels were calculated using the ΔΔCT method within ABI 7500 System Software 2.0.4. All gene expression levels were normalized to the internal standard gene, *Gapdh*. The means and standard error were calculated from triplicate measurements. Significance was determined using the Student’s *t*-test. A *P*-value of less than 0.05 was considered significant, and a *P*-value of less than 0.01 was extremely significant.

### Analysis of Genome-Wide DNA Methylation Level

DNA methylation data were downloaded from published papers (Smallwood et al., 2011; Seisenberger et al., 2012; Liu et al., 2016; Zhang et al., 2016b). Raw data reads were first trimmed of adaptor sequences and low-quality reads. The clean reads were then mapped to the mouse genome (MGSCv37, mm9) assembly using Bowtie2 2.3.4.1. PCR duplicates were removed by Samtools 2.0.1, and the uniquely mapped reads (MAPQ > 10) were kept as the input Bam files. The R packages methylated DNA immunoprecipitation (MEDIPS) and quantitative sequencing enrichment analysis (QSEA) were used to quantify the absolute DNA methylation level.

### Statistical Analysis

All results are shown as mean value and standard deviation (s.d.). All error bars indicate s.d. Prism software was used to perform all statistical analyses of Student’s *t*-test, and *P* values <0.05 were considered significant in all analyses. At least three independent experiments were conducted to draw conclusions. No samples or animals were purposely excluded from analyses.

## Results

### Establishment of Lectin Microarrays for Low-Input Cells

Because it is difficult to obtain a large number of embryos, we first established lectin microarrays for low-input cells using SIM mouse embryo-derived thioguanine and ouabain-resistant (STO) feeder cells. The lectin microarray contained 56 kinds of plant lectins (Supplementary Figure S1), most can only recognize the end of the sugar chain structure, although some can only recognize one sugar group and others can recognize the oligosaccharide structure formed by two or more sugars. Information on the 56 lectins on the chip and the sugar chain structure recognized by the lectins are shown in Supplementary Table S2.

To explore protein loading and sealing conditions of the lectin microarray, we first inferred the best experimental conditions for detecting the glycosylation patterns of glycoproteins of the STO cell lysates. The results showed that the background signal of the microarray sealed with protein-free sealing solution was low, and the intensity and signal-to-noise ratio of the positive signal in the sample were high (Supplementary Figures S2A–C). The microarray sealed by BSA had a higher background signal and a lower signal-to-noise ratio (Supplementary Figures S2D–F). Therefore, the sealing effect of protein-free sealing solution was better than that of BSA. In the following experiments, protein-free sealing solution was used to seal the microarray.

We further studied the signal intensity of lectin binding in different cell lysate concentrations with protein-free sealing. The results showed that, when the sample concentration was 5 ng/μl, the binding of some lectins to sugar chains was not saturated. Additionally, the fluorescence signal value was low because the sample amount was too small, which affected the detection of positive signals (such as ASA, VFA, and black bean crude extract). When the sample concentration was 25 ng/μl, the binding of most lectins to sugar chains was in the linear binding range (such as GNL, calsepa, WFA, CSA, ACL, and GHA) or reached saturation (such as MNA-M, AMA, VVA mannose, and PHA-E). The fluorescence signal values of a small number of lectins were reduced when the sample concentration was 50 ng/μl (such as PSA, LCA, SNA-I, and ConA). No fluorescence signals were detected in some lectins (such as SSA, Tl, malii, Lal, NPL, PWM, GSL I-B4, ABA, and UDA) at sample concentrations of 5, 25, and 50 ng/μl, which may be because the total protein in the lysate did not contain any bound sugar chains (Supplementary Figure S3). Additionally, comparing the results of fluorescence scanning at concentrations of 25 and 50 ng/μl revealed that the experimental results have good repeatability. Therefore, when the sample concentration is 25 ng/μl, there was positive signal detection despite a reduced amount of cell lysate. The following experiments used protein-free blocking solution to block the microarray, and the total protein concentration of cell lysate was 25 ng/μl.

### Dynamic Glycosylation Patterns of Glycoproteins in Mammalian Oogenesis and Preimplantation Embryo Development

**FIGURE 1:**
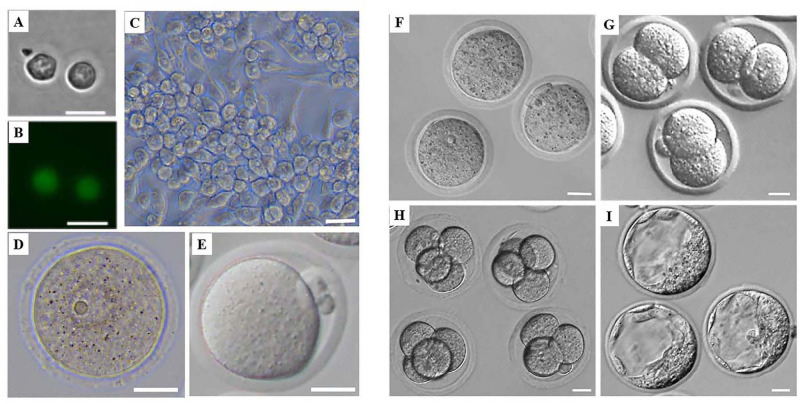
Morphology of mouse female germ cells and preimplantation embryos at different developmental stages. **(A)** Mouse PGCs from Stella-Egfp-IRES-Cre/Ert2 transgenic mice after flow cytometry. **(B)** Mouse PGCs from Stella-Egfp-IRES-Cre/Ert2 transgenic mice under a fluorescence microscope after flow cytometry. **(C)** Mouse FGSCs. **(D)** Mouse GV oocytes. **(E)** Mouse MII oocytes. **(F–I)** Mouse preimplantation of zygotes **(F)**, 2-cell embryos **(G)**, 4-cell embryos **(H)**, and blastocysts **(I)**. Scale bars: 20 μm.

**FIGURE 2:**
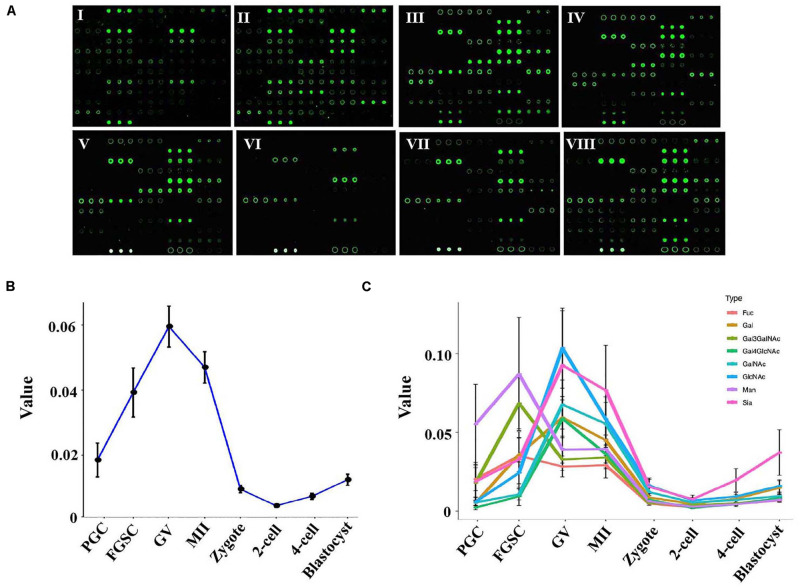
Glycosylation patterns of glycoproteins in female germ cells and preimplantation embryos. **(A)** Representative lectin microarray fluorescence images of mouse female germ cells and preimplantation embryos at different developmental stages. I: PGC; II: FGSC; III: GV oocyte; IV: MII oocyte; V: zygote; VI: 2-cell stage embryo; VII: 4-cell stage embryo; VIII: blastocyst. **(B)** Average lectin signal intensity in the developing female germ cells and preimplantation embryos. **(C)** Average signal intensities of different types of lectins in oogenesis and preimplantation embryos.

The glycosylation patterns of glycoprotein were analyzed using optimized high-throughput lectin microarrays with low-input cells. The lysates of the collected primordial germ cells (PGCs), female germline stem cells (FGSCs), germinal vesicle (GV) oocytes, metaphase II (MII) oocytes, fertilized eggs, 2-cell embryos, 4-cell embryos, and blastocyst-stage embryos (Figure 1) were allowed to react with the lectin microarrays. The microarrays were scanned for measuring the interations. The microarray scanning results (Figure 2A) indicated a low background signal of the microarrays and a high the signal-to-noise ratio of the positive signal in the samples.

The original data of the microarrays were imported into the data processing software (GenePix Pro 6.0) to obtain the fluorescence signal intensity of all lectins. Then, the maximum normalization method was used to normalize the data of different lectin microarrays, and the relative fluorescence signal intensity was obtained. The lectin-binding spectrum of the lysate of female germ cells and preimplantation embryos at different developmental stages are shown in Supplementary Figure S4.

To study the dynamic changes in the glycosylation levels during the different developmental stages of oogenesis and preimplantation embryos, we compared the total lectin signal intensity in these stages. A dramatic increase in the average lectin signal intensity occurred in the PGCs and GV oocytes; the average signal intensity increased from 0.019 in PGCs to 0.040 in FGSCs and further to 0.059 in the GV oocytes. However, the total lectin signal intensity dramatically decreased as the GV oocytes developed into mature MII oocytes and then into fertilized eggs; the average total lectin signal intensity decreased from 0.059 in the GV oocytes to 0.046 in the MII oocytes and further to 0.009 in zygotes. Further reduction of total lectin signal intensity to around 0.004 occurred in the transition from zygotes to the 2-cell stage. Later, during the development of 2-cell into the blastocyst stage, the total lectin signal intensity was always low but showed a slowly increasing trend; the average total lectin signal intensity increased from 0.004 in the 2-cell stage to 0.007 in the 4-cell stage and further to 0.012 in the blastocyst stage (Figure 2B).

These results indicated that the glycosylation level of proteins in PGCs tended to linearly increase during the differentiation from PGCs to GV oocytes, and the glycosylation of proteins may be very important for PGC and FGSC differentiation. However, during oocyte maturation and fertilization (from GV oocytes to MII oocytes and then to fertilized eggs), glycosylation level gradually decreased. Protein glycosylation in preimplantation embryos was low.

We then studied the dynamic changes occurring in the different types of lectins. The results showed that most types of lectins showed the same dynamic trend except for Man-binders and Gal3GalNAc-binders (Figure 2C). Man-binders and Gal3GalNAc-binders had the highest lectin signal intensity in FGSCs, which indicated that these two types of lectins play an important role in the self-renewal and differentiation of FGSC. We also found that Sia-binders increased more dramatically than other types of lectins during embryonic development; these indicated their importance in embryonic development.

### Differences in Glycosylation Patterns of Glycoproteins in Mammalian Oogenesis and Preimplantation Embryo Development

**FIGURE 3:**
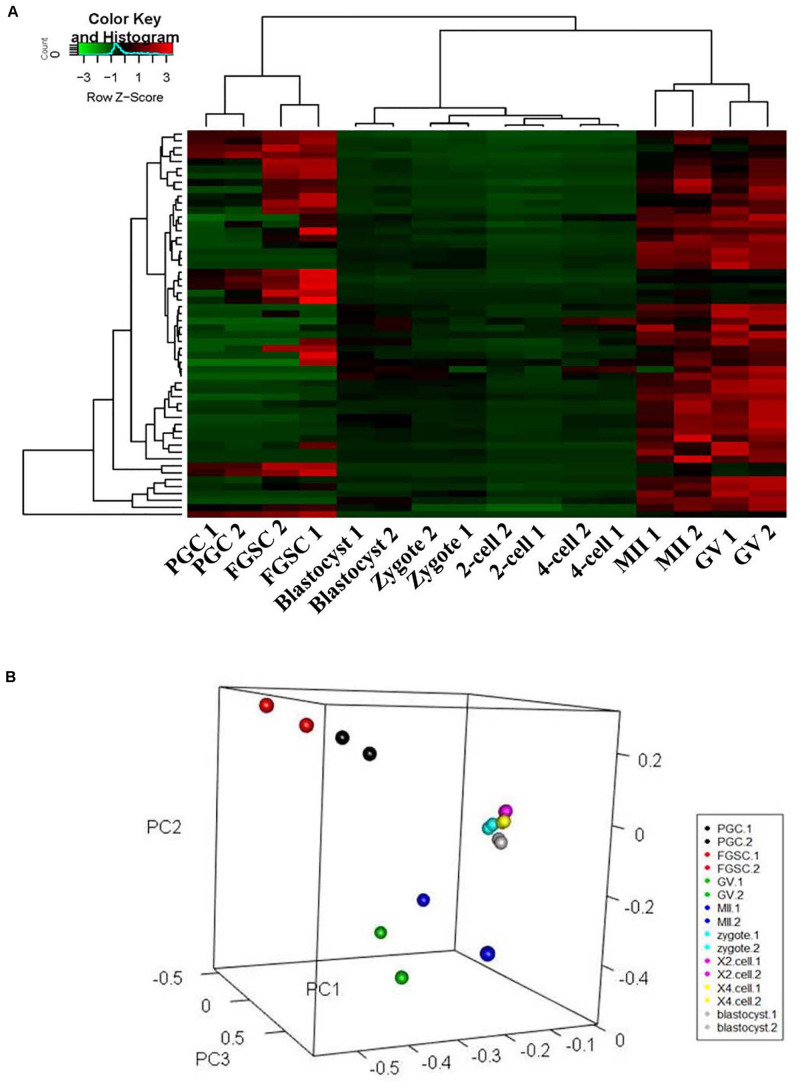
Cluster and principal component analysis of glycosylation patterns of glycoproteins in female germ cells and preimplantation embryos. **(A)** Unsupervised clustering of the glycan profiles of mouse female germ cells and preimplantation embryos at different developmental stages. **(B)** Principal component analysis in mouse female germ cells and preimplantation development.

To classify the female germ cells and preimplantation embryos from different developmental stages based on the characteristics of their patterns of glycoproteins, unsupervised cluster analysis and principal component analysis (PCA) were carried out for the entire normalized lectin-binding spectral data. The results of unsupervised cluster analysis are shown in Figure 3A; it shows that the glycosylation patterns of glycoproteins for the eight types of cells clustered into three groups, in which PGCs and FGSCs; GV and MII oocytes; and fertilized eggs, 2-cell embryos, 4-cell embryos, and blastocysts clustered together because of similarity in their glycosylation patterns. The classification of the glycosylation patterns of glycoproteins of eight types of cells by PCA are consistent with those of unsupervised cluster analysis (Figure 3B).

**FIGURE 4:**
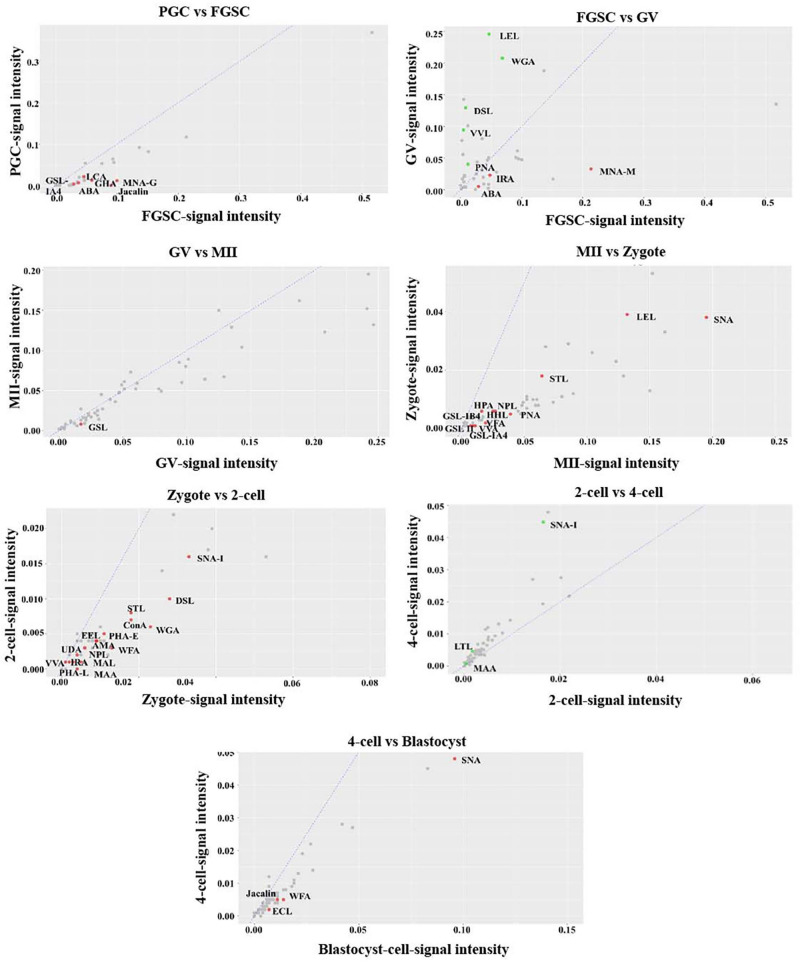
Variation in the characteristics of glycan profiles of cells in adjacent developmental stages. The lectin-binding patterns of adjacent developmental stages are compared to identify the different binding lectins (fold change ≥1.5, *P* < 0.05).

For further investigation, the glycosylation patterns of glycoproteins from cells of adjacent developmental periods were compared and different binding lectins were found (fold change ≥1.5, *P* < 0.05; Figure 4). Several lectins differed between FGSCs and GV oocytes, MII oocytes and fertilized eggs, and fertilized eggs and 2-cell-stage embryos. This indicated that protein glycosylation changed remarkably during the transition of these three developmental stages. Specifically, there were eight kinds of lectins with significant differences between FGSCs and GV oocytes. The signal intensities of three of these lectins were higher in FGSCs than in the GV oocytes: MNA-N for Man recognition, ABA for Galβ3GalNAc recognition, and IRA for GalNAc recognition. This indicates that these three glycosylation types may be involved in the maintenance of FGSC characteristics. The signal intensities of the remaining five lectins were lower in the FGSCs than in the GV oocytes: PNA for terminal βGal recognition; LEL, DSL, and WGA for GlcNAc recognition; and VVL for GalNAc (O-type) recognition. These results demonstrate that these five types of glycosylation may be involved in the regulation of FGSC differentiation.

It is worth mentioning that there were 12 kinds of lectins with significant differences between MII oocytes and fertilized eggs, and the signal intensities of these 12 lectins were higher in MII oocytes than fertilized eggs. These 12 lectins included: VFA, VVA mannose, HHL, and NPL, which recognized Man; GSL-IB4 and PNA, which recognized Gal; SNA, which recognized Siaα2-6Gal; GSL II, STL, and LEL, which recognized GlcNAc; and GSL-IA4 and HPA, which recognized GalNAc. The results showed that the glycosylation levels were lower in the above five types in fertilized eggs than MII oocytes. After fertilization, MII oocytes formed fertilized eggs with totipotency. During this process, the glycosylation levels of the above five types were reduced. However, whether the decrease of glycosylation levels of these five types was related to recovery of zygote totipotency needs to be studied further.

In addition, compared with 2-cell embryos, the signal intensities of 15 kinds of lectins were higher in fertilized eggs: MAL I for Galβ4GlcNAc; WFA for GalNAc; UDA, DSL, WGA, STL, and LEL for GlcNAc; VVA, AMA, ConA, and NPL for Man; PHA-E for the complex sugar chain structure Galβ4GlcNAcβ2Manα6(GlcNAcβ4 (GlcNAcβ4Manα3)Manβ4; SNA-I for Siaα2-6GalNAc; LCA for Fucα1-6-GlcNAc; and RCAI for Gal. This finding indicated that the glycosylation levels of the above eight types of lectins decreased when the fertilized egg developed into the 2-cell stage. During the development of female germ cells and blastocysts after fertilization, most types of lectins associated with glycosylation modification occurred during development from fertilized eggs into 2-cell embryos, which indicated that protein glycosylation might play an extremely important role in transformation during this development period.

### Validation of Lectin Microarrays by Glycosyltransferase Gene Expression Analysis

Protein glycosylation is catalyzed by glycosyltransferase. We downloaded transcriptome data of female germ cells and preimplantation embryos at corresponding developmental stages from NCBI^1^, and further analyzed the expression changes of some glycosyltransferase genes. The results showed that the changes in protein glycosylation among different developmental stages were basically consistent with the expression changes of glycosyltransferase (Supplementary Figure S5). For example, the signal intensities of VVA mannose, VFA, HHL, and NPL, which recognized Man, were significantly higher in MII oocytes than fertilized eggs. The expression levels of the mannosyltransferase genes Alg1 and Alg2 were significantly higher in MII oocytes than fertilized eggs. Similarly, the signal intensity of SNA-I, which recognized sia2-6GalNac, was significantly higher in 4-cell embryos than 2-cell embryos. The expression level of St6galnac2 was also significantly higher in 4-cell embryos than 2-cell embryos (Supplementary Figure S5).

The change of glycosylation level detected by the lectin microarrays was basically consistent with changes in expression levels of the corresponding glycosyltransferase gene, which further demonstrated that the detection results of the lectin chip obtained in this experiment reliably reflected the glycosylation change of the female germ cells and the corresponding development period of preimplantation embryos.

### LEL Was Involved in Mammalian Oogenesis and Preimplantation Embryo Development

**FIGURE 5:**
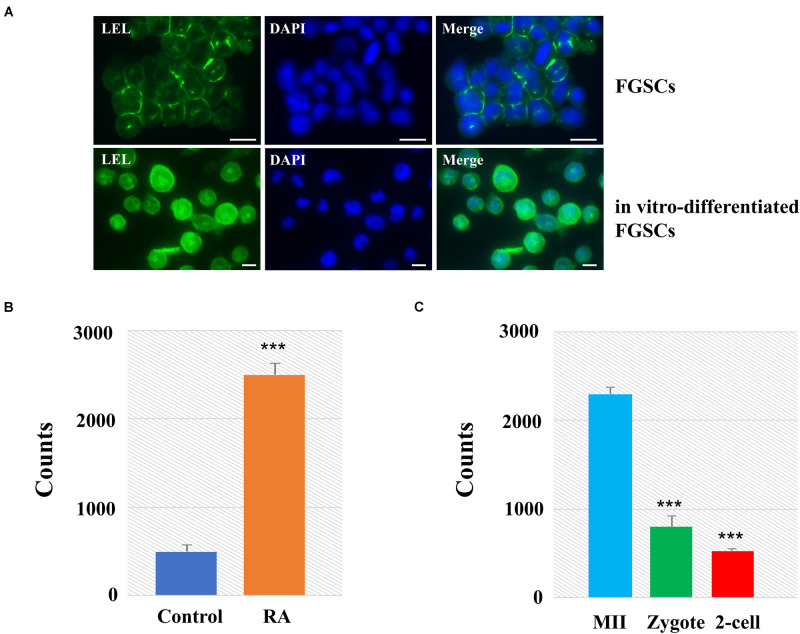
LEL was involved in mammal oogenesis and preimplantation embryo development. **(A)** The FGSCs was detected by lectin-based immunofluorescence analysis with the fluorescein LEL. **(B)** Analyses of glycosylation patterns of LEL in *in vitro*-differentiated FGSCs and FGSCs by lectin-based immunosorbent assays. **(C)** Analyses of glycosylation patterns of LEL in MII oocytes, zygotes and 2-cell embryos by lectin-based immunosorbent assays. ***P < 0.01. Scale bars: 10 μm.

Among these lectins with significant differences, we found that LEL involved not only in oogenesis but also in preimplantation embryo development. We then applied lectin-based immunofluorescence assays to the analyses of glycosylation patterns of LEL during the differentiation of FGSCs induced by retinoic acid and granulosa cells (Ding et al., 2016; Zou et al., 2018). The results showed positivity for LEL both in FGSCs and *in vitro*-differentiated FGSCs, and the fluorescence intensity is stronger after differentiation (Figure 5A). Next, the developed lectin-based immunosorbent assays showed that the signals from *in vitro*-differentiated FGSCs were elevated compared to FGSCs (Figure 5B). These results showed that LEL was involved in the differentiation of FGSCs and might be a marker of FGSC differentiation.

We then applied lectin-based immunosorbent assays to the analyses of glycosylation patterns of LEL in preimplantation embryo development, and found: (1) the relative abundances of LET in each group were correlated reasonably well with the lectin microarray signals; (2) the signals from MII oocytes were elevated compared zygotes and 2-cell embryos (Figure 5C). These results revealed that LEL plays an important role in the process of fertilization and subsequent embryonic development.

### Dynamic TET Analyzed in Mammalian Oogenesis and Preimplantation Embryo Development Using a Bare Gold Electrode Modified With PANI/AuNPs

**FIGURE 6:**
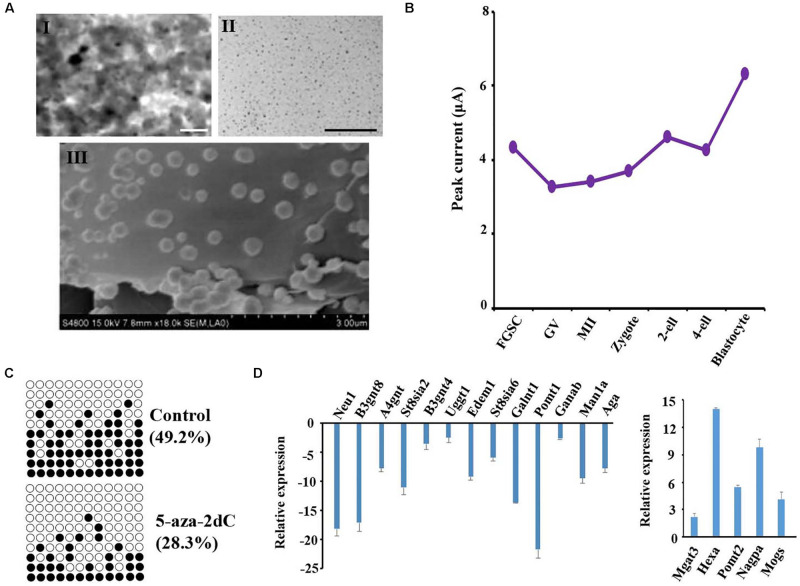
Glycosylation patterns of glycoproteins are related to DNA methylation. **(A)** Transmission electronic microscope (TEM) images of the polyaniline/AuNPs (I), AuNPs (II), and scanning electron microscope (SEM) images of polyaniline (III). **(B)** Peak currents of the TET1 protein across female germ cells and preimplantation embryos at corresponding developmental stages. **(C)** Methylation levels at CpG sites in the LINE-1 in treated groups and control. Solid circles: methylated CpG sites; hollow circles: unmethylated CpG sites. **(D)** Gene expression analysis is performed using the transcriptome data from NCBI of female germ cells and preimplantation embryos at corresponding developmental stages. Scale bars: 200 nm.

Previous studies reported that the TET protein family, which includes important enzymes of DNA demethylation, is regulated by *O*-glycosylation, and plays an important role in maintaining the stemness of stem cells (Zhang et al., 2014; Hahn et al., 2019; Ross and Bogdanovic, 2019; Yu et al., 2019). The dynamic protein levels of TET were investigated by electrochemical techniques because of their high sensitivity, low instrumentation cost, relatively shorter analysis times, and lack of tedious extraction procedures when compared with other analytical techniques (Pandit et al., 2017). The size and shape of the prepared PANI and AuNPs were examined by a scanning electron microscope. The results confirmed that the AuNPs adhered to the top of PANI and the rough exterior provided a large surface area for the adsorption of the target species at the modified electrode (Figure 6A). The nanomaterials were then modified on a bare gold electrode using the layer-by-layer assembly technology; the redox peak potential was higher for the PANI/AuNPs than PANI and the bare gold electrode (Supplementary Figure S6). These results indicated that the nanocomposite PANI/AuNPs improved the sensitivity of electrode signal capture. The modified nanocomposite was further subjected to accuracy measurements by analyzing the TET protein level of spermatogonial stem cells (SSCs) and FGSCs. The results showed that the TET protein level was similar in SSC and FGSCs (Supplementary Figures S7A,B). When analyzing the TET protein level of in mammal oogenesis and preimplantation embryo development, we found the TET protein level decreased almost linearly during the differentiation of FGSCs into GV oocytes, and slowly decreased during the development of GV oocytes into mature MII oocytes. After fertilization, the TET protein level increased and peaked at the blastocyst stage. The trend in the variations of the TET protein level was opposite to that of the glycosylation patterns of glycoproteins in oogenesis and preimplantation embryo development (Figure 6B and Supplementary Figures S7B–H).

### Correlation Between Glyco-Gene Expression and DNA Methylation in Female Germline Stem Cells

To further study the impact of epigenetic changes on the glycosylation pattern of the glycoproteins, FGSCs were treated with the DNA methyltransferase inhibitor 5-aza-2′-deoxycytidine (5-aza-2dC). Prior to glycosylation pattern analysis, the treatment efficiency was checked by pyrosequencing of the long interspersed nuclear elements (LINE-1). Pyrosequencing analysis of the LINE-1 showed a significant decrease in the CpG methylation after treatment with 1 μM of 5-aza-2dC (Figure 6C). Subsequently, quantitative reverse transcription PCR (qRT-PCR) was employed to analyze the effects of 5-aza-2dC treatment on the expression of 30 glyco-genes; these genes included the main glycosyltransferases and glycosidases involved in both N- and *O*-glycosylation (Figure 6D). Considerable differences were detected in the expression levels of 18 genes. Thus, approximately 60% of the glyco-genes were affected by global genome hypomethylation.

### Integrated Analysis of DNA Methylation and Glycosylation Landscapes in Mammalian Oogenesis and Preimplantation Embryo Development

**FIGURE 7:**
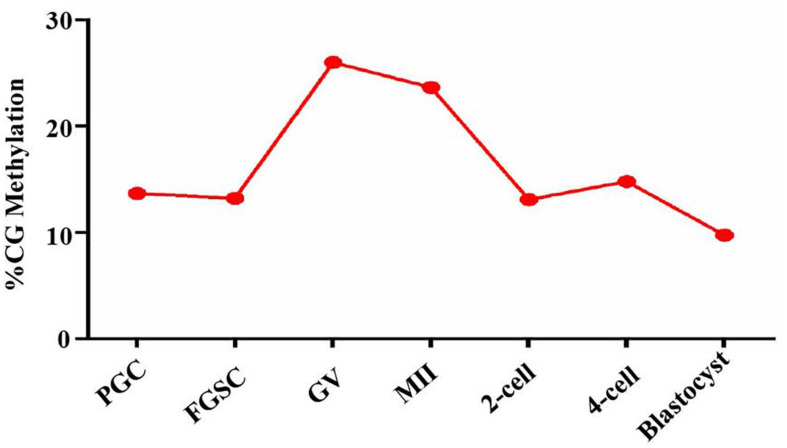
DNA methylation level in oogenesis and preimplantation embryo development. Methylation landscape across each stage of female germ cells and preimplantation embryos. The averaged DNA methylation level of each developmental stage is calculated based on the overlapped 100-base-pair (bp) tiles detected in all of the developmental stages analyzed.

Based on the above results that proved the relation between glycosylation patterns of glycoproteins and DNA methylation, we carried out a genome-wide DNA methylation analysis during oogenesis and preimplantation embryo development. From the NCBI, we downloaded DNA methylation datasets of female germ cells and preimplantation embryos at corresponding developmental stages, and further analyzed the DNA methylation level (Figure 7). The PGCs displayed relatively lower methylation. Following sex determination, the FGSCs also maintained lower methylation. However, methylation increased with differentiation. After fertilization, the methylation decreased until it was the lowest in the blastocyst stage. The trend in variation of DNA methylation was similar to the variation in the glycosylation patterns of glycoproteins, and opposite to the variation in the TET protein level in oogenesis and preimplantation embryo development.

## Discussion

Glycosylation is one of the most important post-translational modifications of proteins and is very important for protein stability, conformational change, and function. More than 50% of proteins in mammalian cells are glycosylated (Apweiler et al., 1999). Many nervous system diseases, immune system diseases, cancer, and even infertility are related to abnormal glycosylation (Pang et al., 2011; Anugraham et al., 2014; Schedin-Weiss et al., 2014). Studies have shown that protein glycosylation is involved in the regulation of basic life processes, such as oocyte development, fertilization, early embryo development, embryo implantation, and organ formation (Dehennaut et al., 2007, 2008; Lay et al., 2013). In this study, high-density lectin microarray technology was used to study the global dynamic changes of glycosylation patterns of glycoproteins during female germ cell and preimplantation embryo development, and provided information that can be used for further study on the protein glycosylation regulation during oogenesis and embryo development.

A microarray with 56 plant lectins was selected. After the microarray was fixed on the carrier substrate, other protein-free sample areas on the carrier substrate were sealed to prevent the tested sample from combining with the substrate and result in a false-positive background elevation. After exploring different sealing conditions, the protein-free sealing liquid was found to produce a good seal.

Because oocytes and preimplantation embryos cannot be expanded and cultured *in vitro* like conventional cell lines, the number of cells that can be obtained at one time is relatively small; therefore, it is relatively difficult to obtain samples. After optimizing the amount of protein samples, we found that the lectin microarray achieved good detection when the protein concentration was 25 ng/μl and the sample volume was 30 μl (i.e., 750 ng total protein). It was reported that the total protein content of each mouse oocyte is about 23 ng (Wassarman et al., 2004). Therefore, based on our optimized experimental conditions, we successfully studied the glycosylation patterns of a small number of oocytes and preimplantation embryos, and successfully solved the limitation of sample source scarcity.

A wide range of glycosylation patterns of glycoproteins in the PGCs, FGSCs, GV oocytes, MII oocytes, fertilized oocytes, 2-cell embryos, 4-cell embryos, and blastocysts were identified that recognized a wide range of glycotypes. Thus, abundant glycotypes existed in the female germ cells and preimplantation embryos. Moreover, the glycosylation type and content of proteins in the developing female germ cells and preimplantation embryos changed with the change in the developmental stages.

Global change of sugar chains is closely related to development. It was previously reported that cells in different developmental states can be classified based on lectin microarray. Toyoda et al. conducted unsupervised cluster analysis on lectin chip data to differentiate the undifferentiated embryonic stem cells and differentiated embryonic stem that formed embryoid bodies (Toyoda et al., 2011). In this study, unsupervised clustering analysis and PCA results showed that PGC and FGSC, GV and MII oocytes, and fertilized eggs and preimplantation embryos had similar carbohydrate profiles, which indicated that protein glycosylation modification and glycosylation content of developing female germ cells and preimplantation embryos had developmental stage specificity and carbohydrate chains. Glycosylation pattern changes were consistent with developmental stage.

It was previously reported that protein glycosylation is involved in regulation of stem cell self-renewal and differentiation. Oct4 and Sox2 in embryonic stem cells undergo *O*-GlcNAc modification. The absence of *O*-GlcNAc modification of these two key proteins will reduce the self-renewal ability of embryonic stem cells (Jang et al., 2012). Wilson et al. studied an immortalized human mesenchymal stem cell (MSc) model and found that *N*-glycosylation and *O*-glycosylation can regulate osteogenic differentiation in the early stage of MSC (Wilson et al., 2018). Silva et al. reported that sialic acid can inhibit human embryonic stem cell differentiation (Alisson-Silva et al., 2014). In this study, we found that the modification levels of Man, Galβ3GalNAc, and GalNAc in FGSCs were higher than those in GV oocytes, which indicates that these three types of glycosylation may be involved in maintaining FGSC characteristics. However, the levels of βGal, GlcNAc, and GalNAc (O-type) glycosylation increased when FGSCs differentiated into GV oocytes, which indicated that the three types of glycosylation may be involved in regulating FGSC differentiation.

Several studies have been conducted the association of glycosylation and methylation. Wahl et al. suggested the presence of an indirect link between DNA methylation and IgG glycosylation that might, in part, reflect environmental exposure (Wahl et al., 2018). Horvat et al. demonstrated that treatment with DNA methyltransferase inhibitors in HeLa cells altered their glycan profiles (Horvat et al., 2013). Fujitaka et al. reported glycosylation and methylation of quercetin and myricetin by cultured cells of Phytolacca Americana (Fujitaka et al., 2017). Dall’Olio et al. highlighted that glycosylation is both under the control of epigenetic mechanisms and is an integral part of the epigenetic code (Dall’Olio and Trinchera, 2017). Klasic et al. showed that DNA hypomethylation up-regulated expression of the MGAT3 gene in HepG2 cells and led to changes in *N*-glycosylation of secreted glycoproteins (Klasic et al., 2016). Chakraborty et al. and Saldova et al. highlighted the role that epigenetics (methylation) plays in glycosylation patterns (Chachadi et al., 2011; Saldova et al., 2011). Hence, a strong link between epigenetics and glycosylation is suggested. Our observations indicated that the trend in the variations of dynamic DNA methylation was similar to the trend of glycosylation patterns of glycoproteins in the female germ cell and preimplantation embryo development.

In conclusion, we established lectin microarrays for low-input cells and performed an integration analysis of the glycosylation patterns of glycoproteins and DNA methylome landscapes in mammalian oogenesis and preimplantation embryo development, and revealed key developmental events with previously unresolved dynamics. Lectin microarrays may become a potent tool for analysis oocytes in search of the association of glycosylation and female infertility, by comparing the glycomes of oocytes between fertile and infertile women. Furthermore, the glycome databases of preimplantation embryos and female germ cells could be apply to evaluated biomarkers related to female germline stem cell and embryonic development. For example, here we have confirmed that LEL was involved in mammalian oogenesis and preimplantation embryogenesis, and might be a marker of FGSC differentiation. Moreover, the precision alteration of protein glycosylation related to oogenesis and early-stage embryos development may provide useful information to find new molecular mechanisms of their development and oocyte and/or early-stage embryos defect related infertility and birth defect therapeutic strategies. Our study not only enriches the glycome databases of preimplantation embryos and female germ cells, but also provides new candidate research clues on protein glycosylation for further understanding the molecular mechanism of sex determination, differentiation, meiosis, gametogenesis, and preimplantation embryo development.

## Data Availability Statement

All datasets generated for this study are included in the article/Supplementary Material.

## Ethics Statement

The animal study was reviewed and approved by Institutional Animal Care and Use Committee of Shanghai.

## Author Contributions

JWa, GT, and XL conducted all the major experiments and data analysis, and wrote the manuscript. YSu and LC performed Lectin microarray acquisition and analysis. YL and WT were responsible for electrochemical technique. YSh performed correlation analysis between glyco-gene expression and DNA methylation in female germline stem cells. XC performed mouse embryo collection. JWu and ST initiated and supervised the entire project, analyzed the data, and wrote the manuscript. All authors reviewed the manuscript and contributed in their areas of expertise.

## Conflict of Interest

The authors declare that the research was conducted in the absence of any commercial or financial relationships that could be construed as a potential conflict of interest.

## Footnotes

1 https://www.ncbi.nlm.nih.gov
