# Supplementary material for: Integrated Glycosylation Patterns of Glycoproteins and DNA Methylation Landscapes in Mammalian Oogenesis and Preimplantation Embryo Development
Source: Front Cell Dev Biol. 2020 Jul 10;8:555. doi: 10.3389/fcell.2020.00555 (PMC7365846; doi:10.3389/fcell.2020.00555)
Supplement: Supplementary file 1 [file Data_Sheet_1.PDF]

Supplemental Information for

Integrated glycosylation patterns of glycoproteins and DNA methylation landscapes in mammal oogenesis and preimplantation embryo development

Jian Wan[1 †], Geng G. Tian[1 †], Xiaoyong Li[1 †], Yangyang Sun[2], Li Cheng[2], Yanfei Li[1], Yue Shen[3], Xuejin Chen[4], Wenwei Tang[5], Shengce Tao[2* ‡], Ji wu[1,3c* ‡]

[*] ***Correspondence***

Ji Wu

jiwu@sjtu.edu.cn

Shengce Tao

taosc@sjtu.edu.cn

[†] *These authors have contributed equally to this work.*

[‡] *These authors have jointly supervised this work.*

Supplementary Information including:

Fig. S1- S7

Table S1- S2

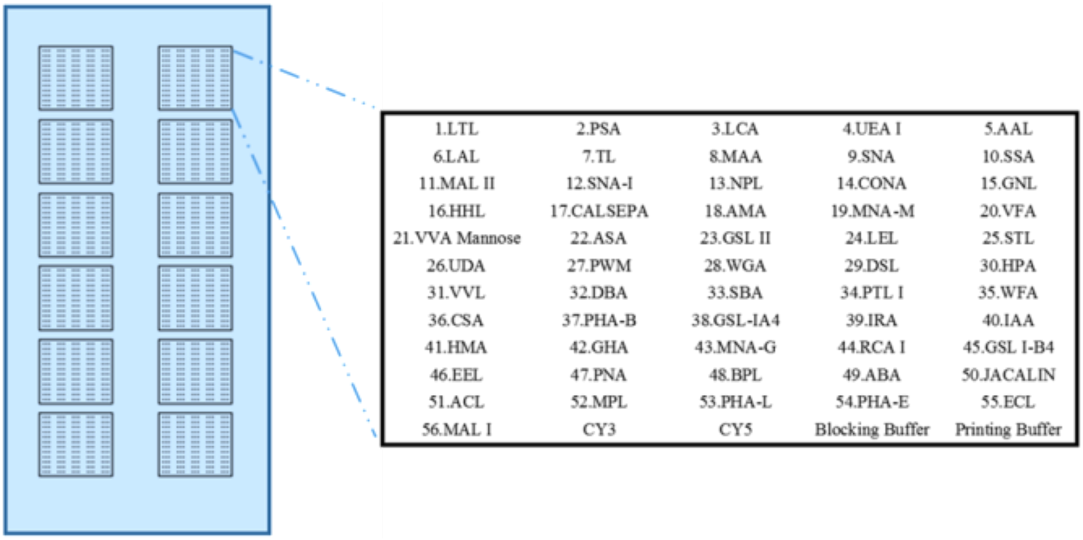

Supplementary Figure 1 | The schematic of lectin microarray.

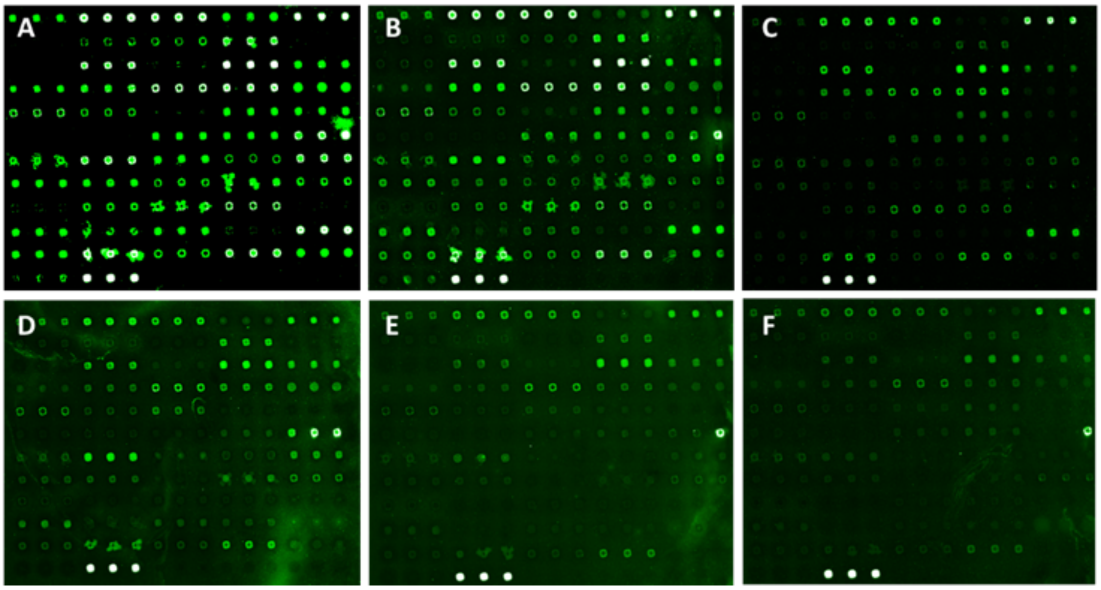

Supplementary Figure 2 | Scanned fluorescence images of lectin microarray with different blocking buffer and sample concentration. (**A-C**) Protein-free blocking. (**D-F**) BSA blocking. A and D: 50 ng/μl; B and E: 25 ng/μl; C and F: 5 ng/μl.

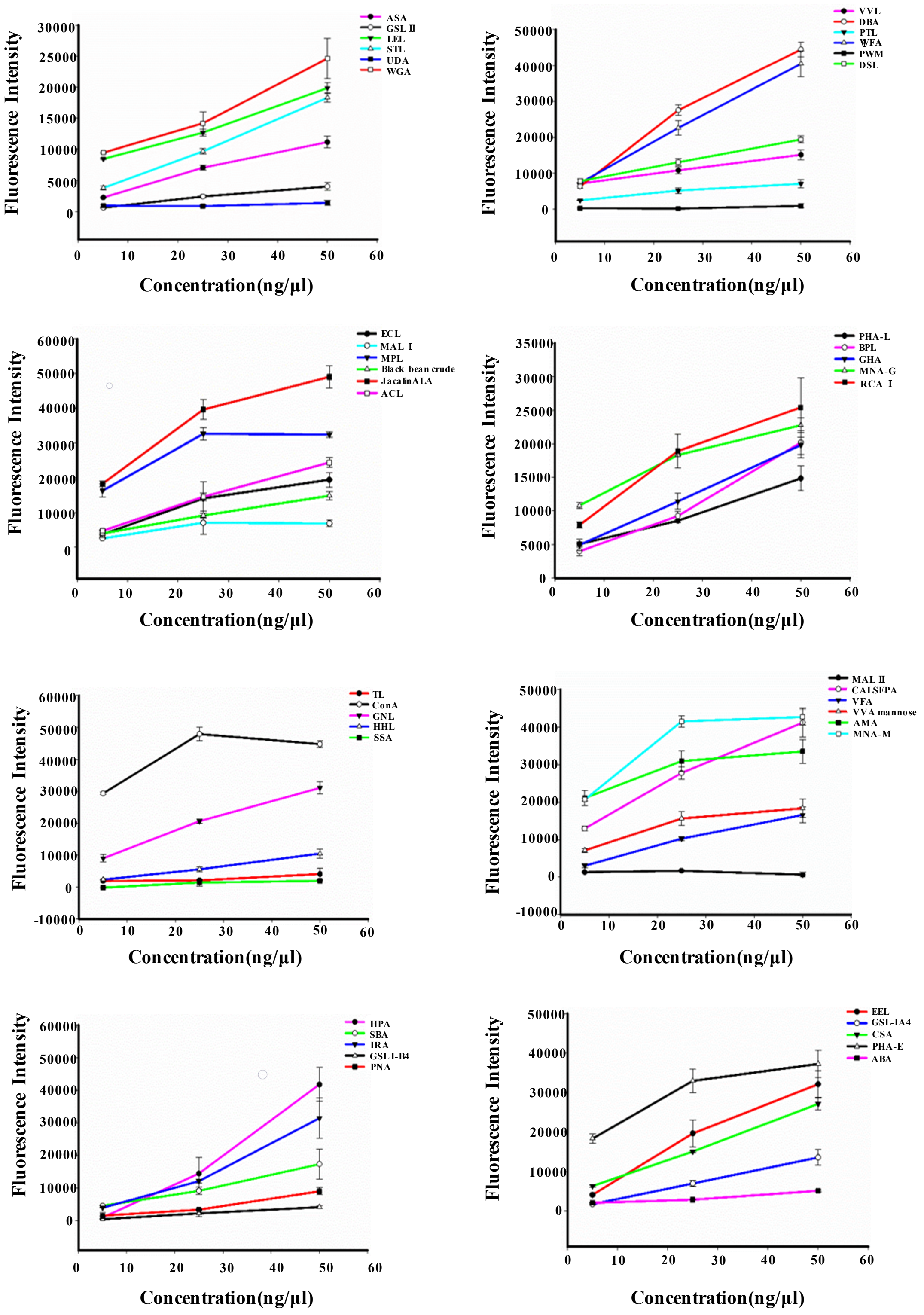

Supplementary Figure 3 | Fluorescence intensity of lectins on the chip under different sample concentration.

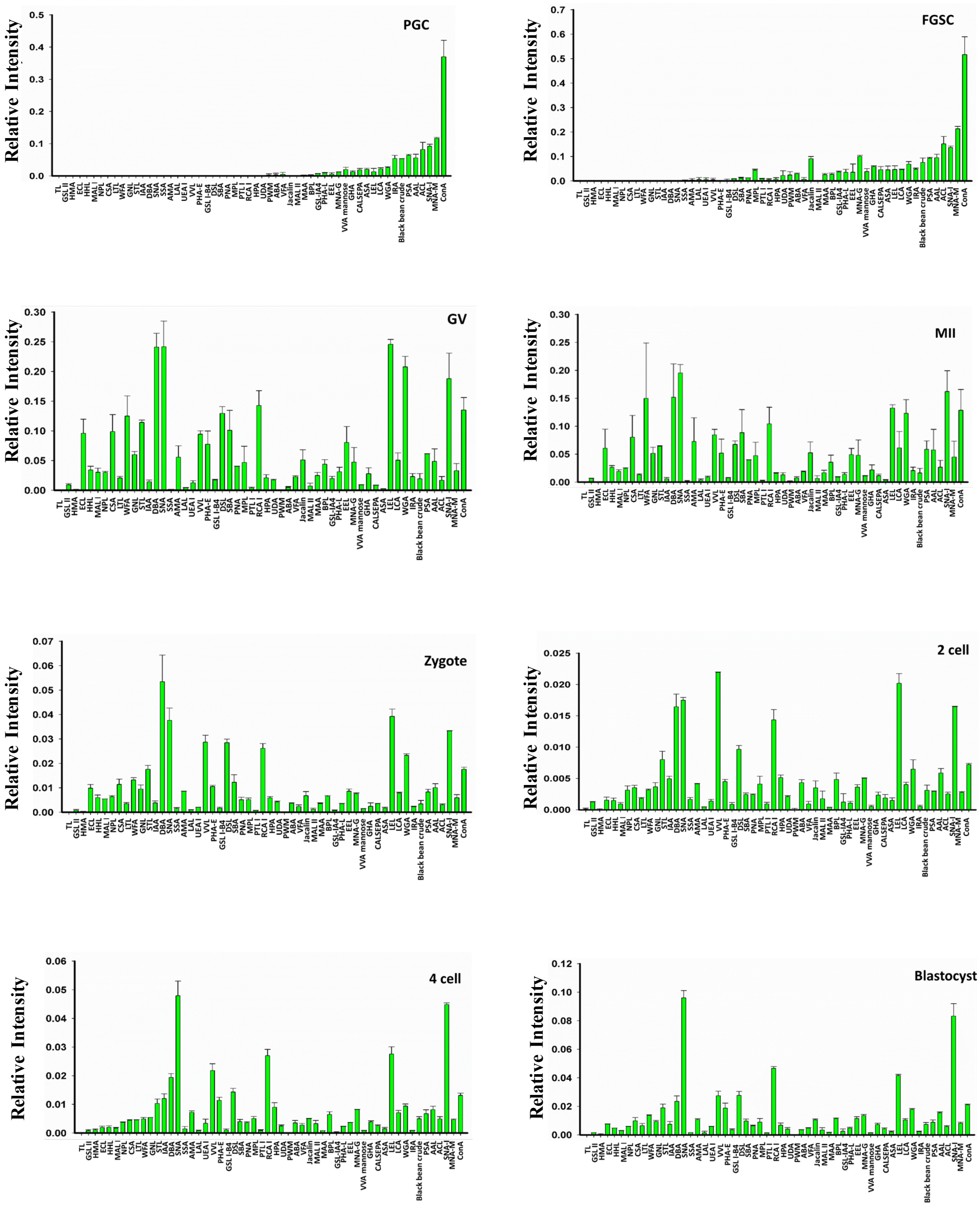

Supplementary Figure 4 | Glycan profiles of mouse female germ cells and preimplantation embryos.

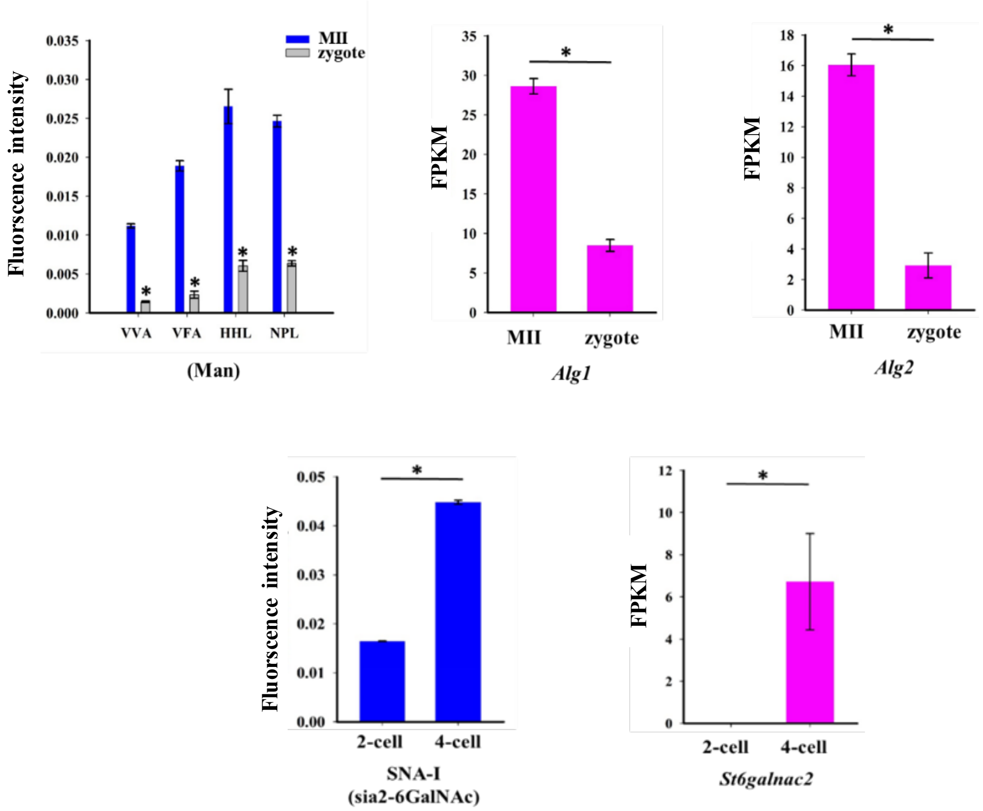

Supplementary Figure 5 | Glycosyltransferase gene expression. Gene expression analysis using qRT-PCR revealed glycogenes that were down- or up-regulated following treatment with 1 μM 5-aza- 2dC. Error bars indicate standard deviation between biological replicates. *P < 0.05.

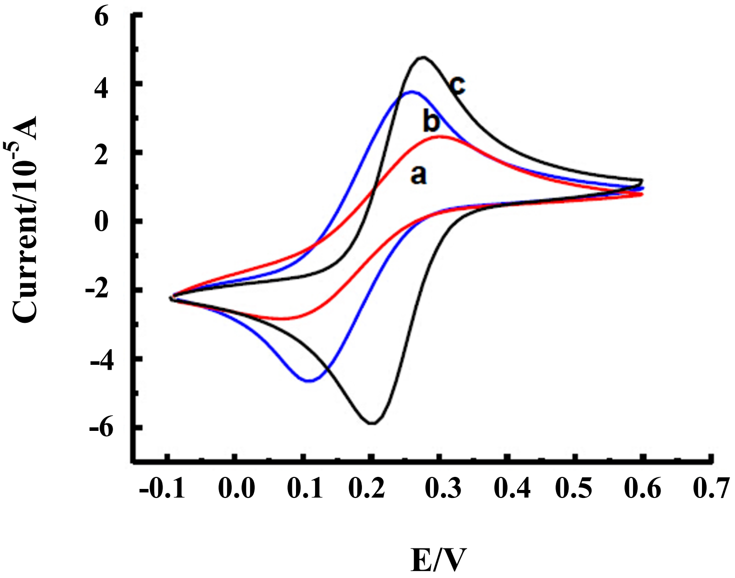

Supplementary Figure 6 | Cyclic voltammograms of bare gold electrode. (a), Polyaniline (b) and Polyaniline/AuNPs (c).

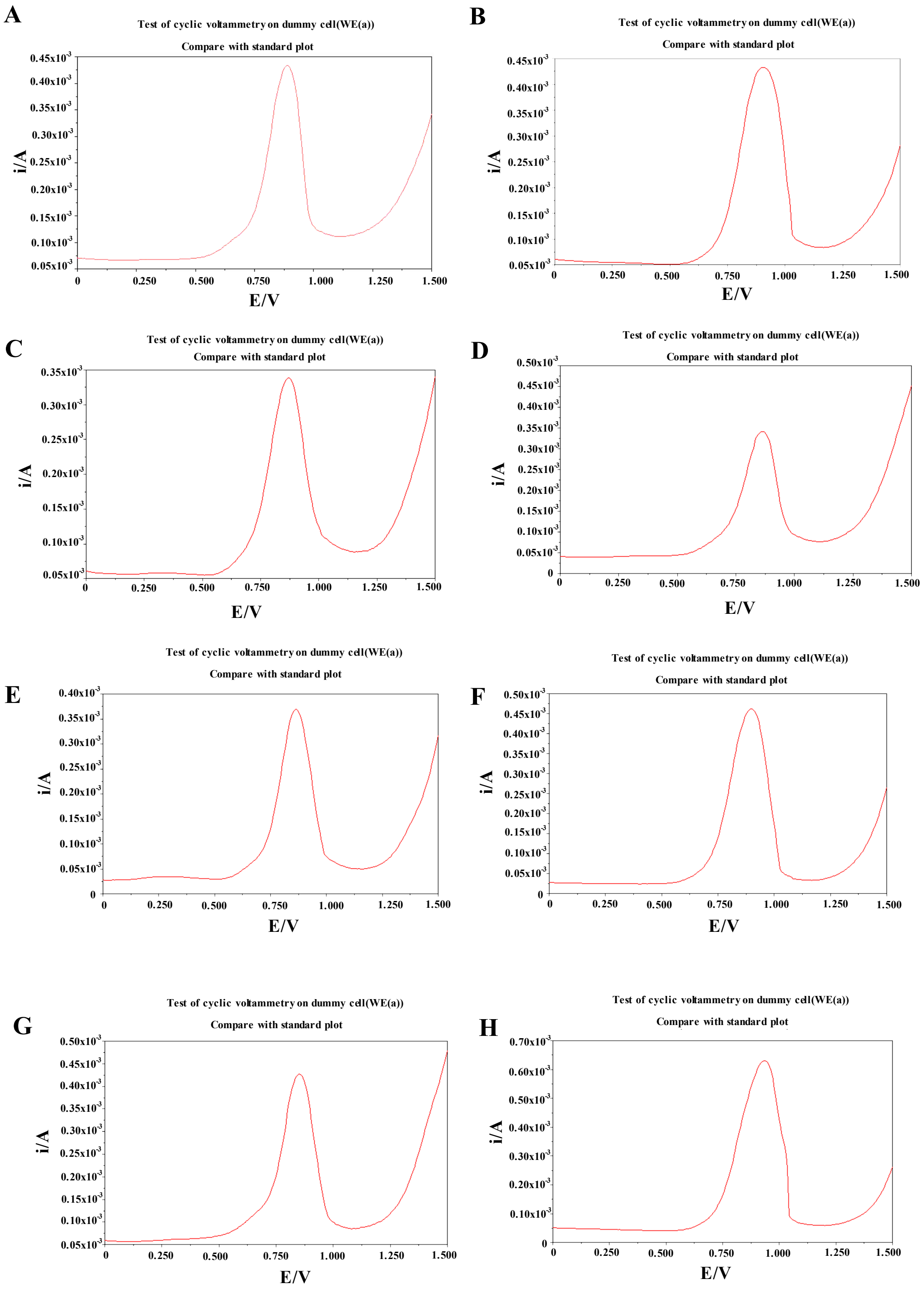

Supplementary Figure 7 | Glycan profiles of mouse female germ cells and preimplantation embryos. Examples of adsorptive stripping voltammograms of TET1 protein across female germ cells and preimplantation embryos at corresponding developmental stages, SSC (A), FGSC (B), GV oocytes (C), MII oocytes (D), Zygote (E), 2-cell stage embryo (F), 4-cell stage embryo (G) and Blastocyte (H).

Supplementary Table 1 Primer details regarding the qRT-PCR analysis

Gene	Product size (bp)	Primer sequence (5’-3’)
Neu1	192	F: GGACCGCTGAGCTATTGGG
		R: CGGGATGCGGAAAGTGTCTA
B3gnt8	198	F: CATCCGAGTCCTGGCTTAAAAA
		R: CAGTCCCCCAAGTCTGACAG
A4gnt	122	F: GCTCACCATGAGGTCTCAGTG
		R: CCACCCTTTCAGAGGTCTCTAT
St8sia2	173	F: AGGCAGAGGTACAATCAGATCA
		R: GAGAGAGCGTCTGGTTGTGTC
B3gnt4	100	F: TCTGAAGGAACGCATACCAGC
		R: AACTGTTAGGTTGGGTGAGCA
Uggt1	120	F: GCCTTACCGTGCCTGTTTTC
		R: GTCTTCTGCTAAGAACTCACTGG
Edem1	180	F: AGTCAAATGTGGATATGCTACGC
		R: ACAGATATGATATGGCCCTCAGT
St8sia6	145	F: TCCTGCGTATGCTCTGGTG
		R: CTGTTCCTGGTGCGTGGTA
Galnt1	131	F: TCCTGCTGCTTTACTTCAGTG
		R: GACGACTGGTTTCCCCATTTC
Pomt1	209	F: GCTGATGCTCATTGAGAACGC
		R: TCCCCATGTATTTGATCCCAACT
Ganab	194	F: TGGCCGTGATGACAACAGTG
		R: GCGCGAGACTAACTTTATCCGA
Man1a	175	F: GGAGCTGGACTGGAAGACAAC
		R: GCAAGCCCCTAAACAGATCCT
Aga	151	F: TTGGCCTTTTAAGAATGCCACT
		R: TTGGCCTTTTAAGAATGCCACT
Mgat3	117	F: ATGAAGATGAGACGCTACAAGC
		R: GGCCAGTTCTCTCGGGAAG
Hexa	155	F: GAGCCACTCTTGACAGAACATC
		R: TGCAAGGACGCTCTCGATTG
Pomt2	154	F: GCAGCACGGAAACTTAAACGG
		R: AGTGAGTCTCATCCCAACAGAT
Nagpa	150	F: TGCTGCCTTACCCACTAGC
		R: CGAAGTGCGACACGAAGGT
Mogs	104	F: CACGTCTATTTCGGCATGAAGA
		R: CACGTCTATTTCGGCATGAAGA
Ogt	108	F: GACGCAACCAAACTTTGCAGT
		R: TCAAGGGTGACAGCCTTTTCA
Glb1	138	F: TGGTGCTCCGCAGGTATCT
		R: TGAAGTCTGGGCCTTTTGTTC

Wbscr17	111	F: CCTGGTGGATGACAACAGTGA<br>R: CCTTCTCGCTTTTGATTGCGTA
Neu4	197	F: CGTGTGCCTGCGTTACTCT<br>R: CAAGGGTTCATAGACCTGTGC
Prkcsh	132	F: ACTACTGCGACTGTAAGGACG<br>R: CACATACCCCATCATTGACCC
Galnt16	162	F: ACCACCACCACCTCTCTCTT<br>R: CGGATCTTCCTCATGGTGGG
Mgat5b	209	F: GAGACCCTTTCGGCTGTTTGT<br>R: CCAGCATATCCATGCGCTTC
C1galt1	105	F: ATGGCCTCTAAATCTTGGCTGA<br>R: AGCCTCTTCTCGCAACAAAATA
Hexb	235	F: CTGGTGTCGCTAGTGTCGC<br>R: CAGGGCCATGATGTCTCTTG
Uggt2	234	F: TGGAAACAGTAAGAGAACTGGCA<br>R: TGCACTTCGTGTGTATAACCAC
St6gal1	152	F: CTCCTGTTTGCCATCATCTGC<br>R: GGGTCTTGTTTGCTGTTTGAGA
Pomgnt1	60	F: GGGCTAGGAAGAAACGGAGC<br>R: CCCGCTGGTTTGTCAGTTTAT

Supplementary Table 2 Fifty-six plant lectins and their recognized sugar chains

NO.	abbreviation	full name	Preferred Sugar specificity
1	LTL	Lotus tetragonolobus lectin	Fucα1-2, Fucα1-3
2	PSA	Pisum sativum agglutinin	Fucα1-6-GlcNAc(N-type), αMan,αGlc
3	LCA	Lens culinaris agglutinin	Fucα1-6-GlcNAc(N-type), αMan,αGlc
4	UEA I	Ulex europaeus agglutinin I	Fucα2Galβ4GlcNAc
5	AAL	Aleuria aurantia lectin	Fucα6GlcNAc, Fucα3, Fucα2
6	LAL	Laburnum anagyroides lectin	Fucα2Galβ4Glc & Fucα2Galβ4Glcβ6GalNAc(β3Gal)
7	TL	Tulipa sp. lectin	N-GalNAc, Gal, Fuc
8	MAA	Maackia amurensis lectin	Siaα2-3Galβ
9	SNA	Sambucus nigra lectin	Siaα2-6Gal/GalNAc
10	SSA	Salvia sclarea	Siaα2-6Gal
11	MAL II	Maackia amurensis lectin II	Siaα2-3Galβ1-4GlaNAc
12	SNA-I	Sambucus nigra	Siaα2-6GalNAc > GalNAc = Lactose
13	NPL	Narcissus pseudonarcissus lectin	αMan(N-type)
14	ConA	Concanavalin A lectin	αMan(N-type),αGlc

15	GNL	Galanthus nivalis lectin	αMan(N-type)
16	HHL	Hippeastrum hybrid lectin	αMan(N-type)
17	Calsepa	Calystega sepiem lectin	Maltose = Mannose > Glucose
18	AMA	Arum maculatum lectin	Man
19	MNA-M	Morniga M lectin	Man
20	VFA	Vicia fava lectin	Man
21	VVA Mannose	Vicia villosa lectin (Mannose)	Man
22	ASA	Allium sativum lectin	Man
23	GSL Ⅱ	Griffonia simplicifolia lectin Ⅱ	α or β GlcNAc(N-type)
24	LEL	Lycopersicon esculentum lectin	(GlcNAc)2-4
25	STL	Solanum tuberosum lectin	(GlcNAc)2-4
26	UDA	Urtica dioica lectin	GlcNAcβ4GlcNAc(N-type)
27	PWM	Phytolacca americana lectin	GlcNAcβ4GlcNAc oligomers & (Galβ4GlcNAc)2
28	WGA	Triticum vulgare lectin	(GlcNAcβ4GlcNAc)1-4 > βGlcNAc > Sia
29	DSL	Datura stramonium lectin	(GlcNAc)2-4, Gal(N-type)
30	HPA	Helix pomation lectin	GalNAc(O-type)
31	VVL	Vicia villosa lectin	GalNAc(O-type)
32	DBA	Dolichos biflorus agglutinin	αGalNAc(O-type)
33	SBA	Glycine max lectin	GalNAc(O-type) > α and βGal
34	PTL I	Psophocarpus tetragonolobus lectin I	Galα3Fucα2Gal, GalNAcα3Fucα2Gal(O-type)
35	WFA	Wisteria floribunda lectin	GalNAc(O-type), GalNAcβ4Galβ4Glc
36	CSA	Cytisus sessilifolius lectin	GalNAc
37	PHA-B	Phaseolus vulgaris sp. lectin	GalNAc > Lactose > Melibiose, Gal, Sia
38	GSL-IA4	Pure Griffonia simplicifolia lectin	Galα3Fucα2Gal(O-type)
39	IRA	lris hybrid lectin	GalNAc
40	IAA	Iberis amara lectin	GalNAc
41	HMA	Homarus americanus lectin	αGalNAc, αFuc, Sia
42	GHA	Glechoma hederacea lectin	Gal, methyl α-D-galactopyranoside, GalNAc
43	MNA-G	Morniga G lectin	Gal
44	RCA I	Ricinus communis agglutinin I	Gal
45	GSL I-B4	GSL I - isolectin B4	Galα3Fucα2Gal(O-type)
46	EEL	Euonymus europaeus lectin	Galα3Gal
47	PNA	Arachis hypogaea lectin	Terminal β-galactose

48	BPL	Bauhinia Purpurea lectin	Galβ3GalNAc
49	ABA	Agaricus bisporus lectin	Galβ3GalNAc
50	Jacalin	Jacalin	Galβ3GalNAc
51	ACL	Amaranthus caudatus lectin	Galβ3GalNAc
52	MPL	Maclura pomifera lectin	Galβ3GalNAc
53	PHA-L	Phaseolus vulgaris leucoagglutinin	Galβ4GlcNAcβ6(GlcNAcβ2Manα3)Manα3
54	PHA-E	Phaseolus vulgaris erythroagglutinin	Galβ4GlcNAcβ2Manα6(GlcNAcβ4)(GlcNAcβ4Manα3)Manβ4
55	ECL	Erythrina cristagalli lectin	Galβ4GlcNAc
56	MAL I	Maackia amurensis lectin I	Galβ4GlcNAc
